# A Brazilian report using serological and molecular diagnosis to monitoring acute ocular toxoplasmosis

**DOI:** 10.1186/s13104-015-1650-6

**Published:** 2015-12-07

**Authors:** Mariana Previato, Fábio Batista Frederico, Fernando Henrique Antunes Murata, Rubens Camargo Siqueira, Amanda Pires Barbosa, Aparecida Perpétuo Silveira-Carvalho, Cristina da Silva Meira, Vera Lúcia Pereira-Chioccola, Ricardo Gava, Plínio Pereira Martins Neto, Luiz Carlos de Mattos, Cinara Cássia Brandão de Mattos

**Affiliations:** Immunogenetics Laboratory, Department of Molecular Biology, Faculdade de Medicina de São José do Rio Preto—FAMERP, Avenida Brigadeiro Faria Lima, 5416, São José do Rio Preto, Sao Paulo state 15090-000 Brazil; Retinopathy Outpatient Clinic, Hospital de Base da Fundação Faculdade Regional de Medicina—HB-FUNFARME, Avenida Brigadeiro Faria Lima, 5544, São José do Rio Preto, Sao Paulo state 15090-000 Brazil; Laboratory of Molecular Biology, of Parasites and Fungi, Instituto Adolfo Lutz—IAL, Aenida Dr Arnaldo,355, São Paulo, São Paulo state 01246-000 Brazil; FAMERP Toxoplasma Research Group, Avenida Brigadeiro Faria Lima, 5416, São José do Rio Preto, Sao Paulo state 15090-000 Brazil; IAL Toxoplasma Research Group, Instituto Adolfo Lutz, Avenida Dr Arnaldo, 355, São Paulo, Sao Paulo state 01246-000 Brazil

**Keywords:** *Toxoplasma gondii*, Ocular toxoplasmosis, Color fundus photography, Optical coherence tomography, Molecular diagnosis, Serology

## Abstract

**Background:**

Toxoplasmosis was recently included as a neglected disease by the Center for Disease Control. Ocular toxoplasmosis is one clinical presentation of congenital or acquired infection. The laboratory diagnosis is being used worldwide to support the clinical diagnosis and imaging. The aim of this study was to evaluate the use of serology and molecular methods to monitor acute OT in immunocompetent patients during treatment.

**Methods:**

Five immunocompetent patients were clinically diagnosed with acute OT. The clinical evaluation was performed by ophthalmologic examination using the Early Treatment Diabetic Retinopathy Study, best-corrected visual acuity, slit lamp biomicroscopy, fundoscopic examination with indirect binocular ophthalmoscopy color fundus photography, fluorescein angiography and spectral optical coherence tomography (OCT). Serology were performed by ELISA (IgA, IgM, IgG) and confirmed by ELFA (IgG, IgM). Molecular diagnoses were performed in peripheral blood by cPCR using the *Toxoplasma gondiiB1* gene as the marker. Follow-up exams were performed on day +15 and day +45.

**Results:**

Only five non-immunocompromised male patients completed the follow up and their data were used for analysis. The mean age was 41.2 ± 11.3 years (median: 35; range 31–54 years). All of them were positive for IgG antibodies but with different profiles for IgM and IgA, as well as PCR. For all patients the OCT exam showed active lesions with the inner retinal layers being abnormally hyper-reflective with full-thickness disorganization of the retinal reflective layers, which assumed a blurred reflective appearance and the retina was thickened.

**Conclusions:**

The presence of IgA and IgM confirmed the acute infection and thus was in agreement with the clinical evaluation. Our results show the adopted treatment modified the serological profile of IgM antibodies and the PCR results, but not the IgG and IgA antibodies and that imaging is a good tool to follow-up patients.

**Electronic supplementary material:**

The online version of this article (doi:10.1186/s13104-015-1650-6) contains supplementary material, which is available to authorized users.

## Background

Toxoplasmic retinochoroiditis is a major cause of posterior uveitis [1–3], it was considered the disease of the year in 2011 [4] and included in the list of neglected diseases by the Centers for Disease Control (CDC) of the United States [5].

*Toxoplasma gondii* infection, the cause of this disease, may also occur during pregnancy during childhood or in adulthood. The clinical symptoms may appear soon after infection or delay with varying degrees of ocular involvement [6, 7]. *T. gondii* form latent cysts directly on the retina, which may be reactivated several years after the primary infection giving rise to retinochoroiditis. Although many episodes of retinochoroiditis are asymptomatic, some result in loss of vision, pain, photophobia, either in isolation or together [8].

For a long time, toxoplasmic retinochoroiditis has been considered the most common eye lesion caused by *T. gondii* infection [9, 10]. Its prevalence appears to be quite variable in different countries, but estimates suggest that from 0.3 to 1 % of Europeans and North Americans develop this disease within 1–2 years of contracting the infection [9, 11, 12]. It is believed that the risk of developing this disease ranges from 18 in 100,000 individuals in the UK to as many as 382 in 100,000 individuals in West Africa [8, 13]. Furthermore, the risk of developing retinochoroiditis among individuals who contracted congenital infection is as high as 20 % before the age of 6 years and that new eye lesions may first appear in adolescence [3, 12, 14–16].

Studies in Brazil have shown that the prevalence of toxoplasma retinochoroiditis is variable but very high in adolescents and adults in some regions of the country; the disease ranges from 2 % in the southeast to 25 % in the southern region [10, 17–20]. A study in the State of Rio Grande do Sul revealed a prevalence of ocular toxoplasmosis of 21.3 % in over 13-year-old individuals and concluded that the disease is a consequence of postnatal infection [10]. In the State of Pernambuco, it was observed that 56.2 % of cases of posterior uveitis were due to *T. gondii* infection [21] and in the State of Rio Grande do Norte, 56.9 % of the patients analyzed in one investigation had bilateral lesions [22]. These studies make it clear that toxoplasmic retinochoroiditis is common in Brazil and thus an analysis of host risk associated with factors of this disease is justified. Furthermore, our recent study showed that the prevalence of toxoplasma retinochoroiditis is approximately 27 % among patients with eye diseases [23].

The growing interest in the investigation of toxoplasmosis has helped to develop strategies for early laboratory diagnosis and clinical intervention [24–30]. Moreover, it has been found that imaging tests such as fundus photography, fluorescein angiography and optical coherence tomography (OCT) help in the assessment, registration and documentation of eye disease, thereby showing whether it is in the acute phase or quiescent, and can also be used to monitor eye involvement in respect to *T. gondii* infection [3, 31–39]. OCT is a noninvasive test that provides data to evaluate morphological changes that occur in the retina, vitreous, and choroid during ocular toxoplasmosis and other ocular diseases. The objective of this study was to monitor the evolution of active ocular disease or acute relapse of ocular toxoplasmosis using serology and molecular tests and evaluate their applicability.

## Methods

### Ethical aspects of the study

This study was approved by the Ethics Committee of the Medicine School in São José do Rio Preto—FAMERP and the selected patients signed informed consent forms after being informed about the nature of the study including the objectives and laboratory procedures that would be performed.

### Patients

In the period between August 2013 and July 2014, patients with clinical suspicion of active lesions or acute relapse of *T. gondii* infection were evaluated. Of a total of 31 patients enrolled and treated, five were selected because they adhered to the treatment regimen and were followed up for a period of 45 days (day 0; day +15;day +45). The and the start of the treatment were performed at day 0. New blood samples were collected for analysis at day +15 and day +45.

The five patients received clinical care and were submitted to all the proposed tests (serological diagnosis of toxoplasmosis, cPCR to detect *T. gondii* in peripheral blood; OCT and fundus photography) at each return consultation.

The inclusion criteria were patients with seropositive samples for toxoplasmosis, scarring of the retina characteristic of toxoplasmosis, and active retinochoroiditis lesion (satellite). Under 18-year-old patients were excluded as were immunosuppressed patients, patients receiving immunosuppression drugs and those with retinochoroiditis with clinical characteristics of other causes.

### Blood sampling

Blood samples were collected in tubes with and without EDTA in all consultations. An investigation of *T. gondii* infection (IgM, IgG and IgA) was made and DNA was extracted from leukocytes.

## Eye exams

### Clinical examination

All patients underwent detailed eye examinations including visual acuity [the logMAR Early Treatment Diabetic Retinopathy Study (ETDRS) chart] with best correction according to standardization recommended by ETDRS [40], measurement of intraocular pressure by Goldmann applanation tonometry, biomicroscopy using a slit lamp, and stereoscopic biomicroscopy performed using a 78 diopters lens (Volk) and classified according to the criteria determined by the ETDRS.

The OCT was performed using the RTVue-100 scanner with an axial image resolution and speed of five axial velocity of 26,000 frames per second using a program to measure the retinal thickness with the cursor placed according to the fixation of the patient or manually in the center of the fovea, when the foveal depression was visible. The analysis strategy utilized the macular thickness map measured in the central region of the retina.

The evaluation was performed using radial cuts (horizontal and vertical of the line and cross line program) within the fundus area encompassing the toxoplasmosis ocular lesion. Whenever possible, three-dimensional OCT was conducted covering the entire retinochoroiditis lesion. In addition, standardized image acquisition programs such as MM5 (5 × 5 mm^2^ horizontal grid 11 by 11 vertical lines with 668 A-scans each and a 3 × 3 mm^2^ grid of six internal vertical and horizontal lines with 400 A-scans each), MM6 (12 radial lines with 1024 A-scans each within 6 mm in diameter) and macula 3D (128 scan lines with 512 A-scans each within 6 × 6 mm^2^) protocols were used, with all checks with signal strength of at least 40 (range 40.4–79.4).

Colored fundus photographs and fluorescent photographs were taken using a digital retinal camera (TRC-50DX, Topcon Medical Systems) in order to document the macula region and optic nerve. Areas of progressive hyperfluorescence (leakage), impregnation of contrast (staining) and transmitted hyperfluorescence (window effect) were observed by fluorescein angiography. The progressive hyperfluorescence with delayed leakage of contrast was considered a sign of lesion activity.

## Therapy regimen

After evaluating the patient at baseline and collecting blood samples, ocular toxoplasmosis was treated using the following protocol: sulfadiazine 1 g four times per day, pyrimethamine 50 mg daily, folinic acid 7.5 mg daily and prednisone 0.5 mg/kg/day for 4 weeks.

## Detection of IgM, IgG and IgA anti-*T. gondii* antibodies

IgM, IgG and IgA anti-*T. gondii* antibodies were investigated using the ELISA test with the ETI—TOXOK-M (IgM), ETI—TOXOK-G (IgG) and ETI—TOXOK-A (IgA) commercial kits (DiaSorin, Italy); the results were confirmed by enzyme-linked fluorescence immunoassay (ELFA) Vidas Toxo IgG and Vidas Toxo IgM (bioMérieux, France) according to manufacturer’s directions.

## Molecular diagnosis of *T. gondii* infection

### Extraction of genomic DNA

Genomic DNA was extracted from 5 mL of peripheral blood samples collected in EDTA as previously described [41] using the commercial QIAamp^®^ DNA Blood Mini Kit (QIAGEN, The Netherlands) .

### Molecular analysis to identify *T. gondii*

#### cPCR

*Toxoplasma gondii* was identified in blood samples using a previously described technique [41] that amplifies a final volume of 25 uL per reaction tube using the reagent GoTaq Hot Start Green Master Mix (Promega, USA). Each reaction tube (mix) contained 25 pmol of each primer, 1 U of Taq DNA polymerase, 10 mM Tris–HCl at pH 8.5, 50 mM KCl, 1.5 mM MgCl_2_, and 200 mM of each dNTP. Two negative controls (ultrapure water and genomic DNA negative for *T. gondii*) and a positive control (DNA extracted from the RH strain of *T. gondii*) were included in each amplification reaction.

The B22 and B23 primers, which amplify a 115-base pair (bp) fragment of the repeat region of the *B1* gene, were utilized [42]. The HGH primers that amplify a 400-bp fragment of the human growth hormone gene were used as a control of amplification and detection of PCR inhibitors. The amplicons were analyzed by electrophoresis in 2 % agarose gel, stained with ethidium bromide and viewed under ultraviolet light.

## Data analysis

Data were analyzed descriptively to determine the importance of OCT to monitor the progression of active ocular disease or acute relapse due to infection by *T. gondii*.

## Results

From the 31 patients enrolled in this study, only five completed the proposed follow up; all of them were male. The mean age was 41.2 ± 11.3 years (range 31–54; median: 35). At the time of inclusion, all of them had positive serology for toxoplasmosis IgG antibodies.

Table 1 presents the data from serological tests for IgM, IgA and IgG anti-*T. gondii* antibodies of the five patients enrolled in this study and Table 2 shows the cPCR results.

**Table 1:** Results of serologic testing by ELISA for IgM, IgA and IgG antibodies of the five patients who completed the follow-up (day 0, day +15, day +45)

Patient	IgM			IgA			IgG		
	D0	D + 15	D + 45	D0	D + 15	D + 45	D0	D + 15	D + 45
Case-01	P	P	P	P	P	P	P	P	P
Case-02	P	N	N	N	N	N	P	P	P
Case-03	N	N	N	N	N	N	P	P	P
Case-04	N	N	N	N	N	N	P	P	P
Case-05	N	N	N	N	N	N	P	P	P

*P* positive, *N* negative

**Table 2:** Results of molecular tests (cPCR) of the five patients who completed the follow-up (day 0, day +15, day +45)

Patient	D0	D + 15	D + 45
Case-01	Negative	Negative	Negative
Case-02	Negative	Negative	Positive
Case-03	Negative	Positive	Negative
Case-04	Positive	Negative	Negative
Case-05	Positive	Negative	Positive

**Fig. 1:**
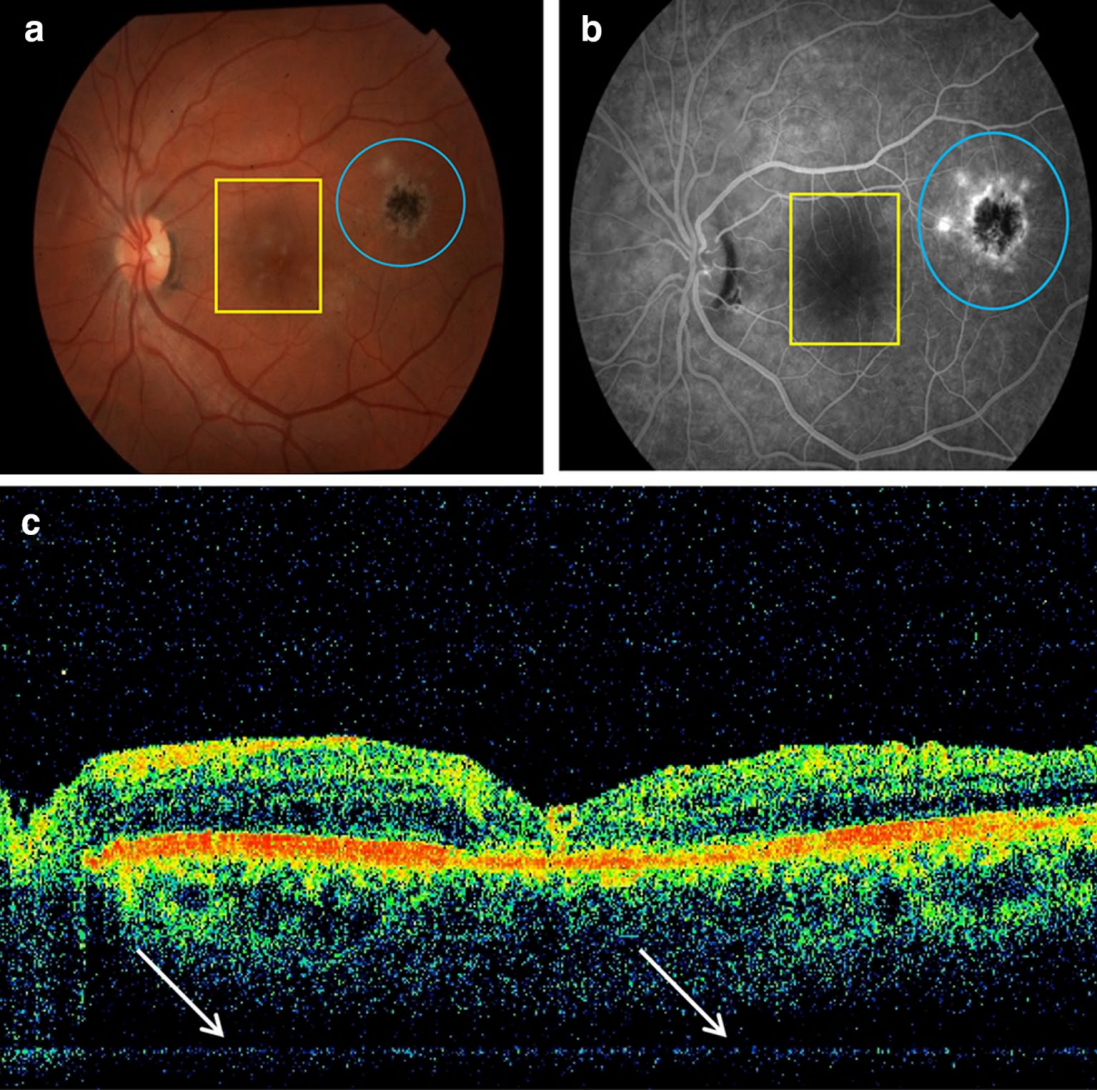
Photodocumentation of pretreatment phase of one patient (case 1). **a** Fundus photography showing a satellite lesion (*yellow square*) of activity suggestive of retinochoroiditis toxoplasmosis in the macula region and a healed retinochoroiditis lesion (*blue circle*); **b** fluorescein angiography showing a satellite lesion suggestive of activity of toxoplasmosis (*yellow rectangle*) in the macula region and a healed retinochoroiditis lesion (*blue circle*); **c** increases in the thickness of the inner retinal layers in perimacular regions (*arrows*) seen by optical coherence tomography

Figure 1 illustrates the results of color retinography imaging (a) fluorescein angiography (b) and OCT (c).

Table 3 shows the pattern of eye involvement of the patients with suspicion of acute ocular toxoplasmosis by color retinography imaging, fluorescein angiography, and OCT.

**Table 3:** Eye involvement of the five patients with suspicion of acute ocular toxoplasmosis

Patient	Acute ocular involvement		Previous scarring	
	Right eye	Left eye	Right eye	Left eye
Case-01	No	Yes	No	Yes
Case-02	Yes	No	No	No
Case-03	NoF	Yes	No	Yes
Case-04	Yes	No	Yes	No
Case-05	No	Yes	No	Yes

Additional informations are described in Additional file 1.

## Discussion

The aim of this study was to evaluate the use of serological, molecular and imaging methods to monitor the status and symptoms of patients clinically diagnosed with acute ocular toxoplasmosis. The *B1* gene of *T. gondii* was used as target for molecular analysis since it has been described as more appropriated to characterize clinical samples in Brazil in contrast with observations from other countries [24, 28, 30, 43–51].

The mean age of the five patients enrolled in this study was about 45-years- old, supporting reports that ocular toxoplasmosis affects young individuals [3, 52, 53]. It is possible that the disease is a result of congenitally acquired infection as the results do not allow us to verify the manner in which the toxoplasmosis was acquired.

The ELISA assay showed that one of the patients (case-01) had acute infection due to the presence of IgM and IgA antibodies at the time of enrollment. This condition remained throughout the follow up which suggests that serological evidence of acute infection may continue even after treatment. This observation is in agreement with literature reports indicating that IgM antibodies persist in patients under treatment for ocular toxoplasmosis. It is also possible that this patient is a special case of individuals who have persistent IgM antibodies (IgM residual); this situation creates additional difficulties in laboratory diagnosis and in the continuation of treatment for long periods of time as it may lead to the impression that an infection is acute or a reactivation of infection [54–60].

By ELISA, one patient (case-02) was positive for IgM antibodies on day 0 but did not have evidence of IgA antibodies in subsequent analyzes. Given that he was positive for IgG antibodies at enrollment in the study, it appears that he seroconverted to a chronic infection. However, these data are not enough to predict the time that the infection occurred. This condition seems to be common in patients infected with *T. gondii* irrespective of clinical evidence of ocular disease [25, 54, 56, 61, 62]. The other patients were not positive for IgM and IgA antibodies at inclusion in the study but the identification of IgG antibodies was used to infer a chronic infection.

Except for one patient (case-01), all responded adequately to the treatment regimen and the imaging tests showed a reduction of ocular inflammation during the follow-up period. This patient shows a different profile since he presented serological evidence of acute infection but the cPCR was negative during the follow up. Maybe this is a case of atypical strain infection not detected by *B1* gene as previously described and the cPCR should be done using other set of primer as being reported in other regions of the world [3, 26, 44, 47, 63–67], but not in South American studies [30, 45, 48–50, 68–72].

There were no variations in the serological profile of three patients (case-03–05) but the cPCR results were positive at day 0 (cases 04 and 05), day 15 (case-03) and day 45 (case-05). These conditions of infection revealed by cPCR in the peripheral blood may be the result of two possible situations: the presence of residual DNA or a false-positive result [25, 43, 48, 56]. These data are in agreement with that reported by Novais et al. 2014 [49] which found positive PCR in Brazilian patients presenting inactive toxoplasmic retinochoroidits lesions and with our previous report [41]. Additionally our study observed that one patient (case-05) had clinical evidence of reactivation of ocular disease based on the PCR results.

Fluorescein angiography showed progressive hyperfluorescence with delayed leakage of contrast and OCT showed that the locations of the lesions of all patients had abnormal inner layers of the retina with hyper-reflective thickened and blurred areas. The level of resolution of this imaging method is well suited to show the characterization of ocular lesions, including those resulting from *T. gondii* infection [36, 39, 73].

The presence of specific antibodies against *T. gondii* (IgM and IgA) identifies acute infection and confirms the clinical evaluation. Furthermore, our data suggest that the treatment used in this study may modify the serological profile of IgM antibodies and the result of cPCR, but not the serological profile of IgG and IgA antibodies. Exceptions to this observation can be seem among patients with tendency to remain with residual IgM specific antibodies [25, 49, 56, 58, 59, 74, 75].

The present investigation was limited by the small number of patients evaluated and included. Would be desirable studies with a large amount of patients around the world and in Brazil which could confirm the results reported here.

## Conclusion

In conclusion, blood tests are useful to monitor ocular toxoplasmosis and to determine whether the infection is acute or chronic. Molecular analysis by PCR helps to identify possible parasitemia and monitor the effectiveness of treatment as therapy together with the immune response should eliminate parasites circulating in the peripheral blood. Finally, this study shows that imaging tests are excellent noninvasive tools of photo documentation and to monitor inflammation and subsequent scarring of areas damaged by the parasite during ocular toxoplasmosis.

## Additional file

10.1186/s13104-015-1650-6 Clinical data and results of imaging studies (fundus photography, fluorescein angiography and Optical coherence tomography) of the five patients who correctly completed the follow-up.

## Abbreviations

CDC: Center for Disease Control
OT: ocular toxoplasmosis
*T. gondii*: *Toxoplasma gondii*
IgG: immunoglobulin G
IgM: immunoglobulin M
IgA: immunoglobulin A
cPCR: conventional polymerase chain reaction
ELFA: enzyme-linked fluorescente assay
ELISA: enzyme-linked immunosorbent assay
OCT: optical coherence tomography
FAMERP: Faculdade de Medicina de São José do Rio Preto
IAL: Instituto Adolfo Lutz
