# Supplementary material for: A Brazilian report using serological and molecular diagnosis to monitoring acute ocular toxoplasmosis
Source: BMC Res Notes. 2015 Dec 7;8:746. doi: 10.1186/s13104-015-1650-6 (PMC4671220; doi:10.1186/s13104-015-1650-6)
Supplement: Supplementary file 1 — 10.1186/s13104-015-1650-6 Clinical data and results of imaging studies (fundus photography, fluorescein angiography and Optical coherence tomography) of the five patients who correctly completed the follow-up. [file 13104_2015_1650_MOESM1_ESM.docx]

**Additional file 1:** Clinical data and results of imaging studies (fundus photography, fluorescein angiography and Optical coherence tomography) of the five patients who correctly completed the follow-up

| **Patient** | **Day 0** | **Day 15** | **Day 45** |
| --- | --- | --- | --- |
| **CASE-01** |  |  |  |
| **Symptoms** | Reduced vision of left eye | Partial improvement of symptoms | Reported significant improvement in visual acuity |
| **Visual acuity** | Right eye = 20/20  Left eye = 20/60 | Right eye = 20/20  Left eye = 20/40 | Right eye = 20/20  Left eye = 20/25 |
| **Tonometry** | Right eye = 14mmHg  Left eye = 14mmHg | Right eye = 14mmHg  Left eye = 14mmHg | Right eye = 14mmHg  Left eye = 14mmHg |
| **Anterior segment Biomicroscopy** | Cells in the anterior chamber 1+/4+  (fine keratic precipitates) | No cells in the anterior chamber | No cells in the anterior chamber |
| **Posterior segment Biomicroscopy** | Vitreous cells 1+/4+  Vitreous haze 1+/4+ | No vitreous cellularity inflammation  No vitreous haze | No vitreous cellularity inflammation  No vitreous haze |
| **Fundus photography** | Healed retinochoroiditis lesion temporal to the macula region and active satellite punctate lesions in the central macular region | Healed retinochoroiditis lesion temporal to the macula region and healing satellite punctate lesions in the central macular region | Healed retinochoroiditis lesion temporal to the macula region and healed satellite punctate lesions |
| **Fluorescein angiography** | Blocked hypofluorescence corresponding to healed lesions and progressive hyperfluorescence with delayed leakage of contrast corresponding to active lesions | Blocked hypofluorescence corresponding to healed lesions and with discreet ‘staining’ corresponding to satellite lesions | Blocked hypofluorescence corresponding to healed lesions and areas of satellite lesions without delayed leakage or ‘staining’ of the contrast corresponding to active lesions |
| **Optical coherence tomography (OCT)** | Increased retinal thickness corresponding to active lesions in the perifoveal region of the inner retina with no signs of significant accumulation of intra-or subretinal fluid | Reduced thickness of the lesions compared to the previous examination (baseline) with no signs of significant accumulation of intra- or subretinal fluid | Small area of hyper-reflectivity probably due to hyperplasia of the retinal pigment epithelium with no signs of intra or subretinal fluid |
| **CASE-02** | **Day 0** | **Day 15** | **Day 45** |
| **Symptoms** | *Floaters* in the right eye | Partial improvement of symptoms | Reported significant improvement in visual acuity |
| **Visual acuity** | Right eye = 20/30  Left eye = 20/20 | Right eye = 20/25  Left eye = 20/20 | Right eye = 20/20  Left eye = 20/20 |
| **Tonometry** | Right eye = 14mmHg  Left eye = 14mmHg | Right eye = 14mmHg  Left eye = 14mmHg | Right eye = 14mmHg  Left eye = 14mmHg |
| **Anterior segment Biomicroscopy** | Cells in the anterior chamber 1+/4+  (fine keratic precipitates) | No cells in the anterior chamber Reduction in keratic precipitates | No cells in the anterior chamber No keratic precipitates |
| **Posterior segment Biomicroscopy** | Vitreous cells 1+/4+  Vitreous haze 1+/4+ | No vitreous cellularity inflammation  Vitreous haze 1+/4+ | No vitreous cellularity inflammation  No vitreous haze |
| **Fundus photography** | Active peridiscal retinochoroiditis lesion in the right eye without signs of macular involvement | Peridiscal retinochoroiditis lesion in the right eye with signs of healing and without macular involvement | Apparently healed peridiscal retinochoroiditis lesion in the right eye without sings of macular involvement |
| **Fluorescein angiography** | Progressive hyperfluorescence with delayed leakage of contrast corresponding to active peridiscal lesion | Delayed discreet ‘staining’ characteristic of healing of lesion | Blocked hypofluorescence corresponding to lesion without areas of delayed leakage or ‘staining’ of the contrast |
| **Optical coherence tomography (OCT)** | Increased retinal thickness corresponding to active lesions in the peridiscal region with no signs of significant accumulation of intra-or subretinal fluid | Reduced retinal thickness compared to the previous examination (baseline) with no signs of significant accumulation of intra- or subretinal fluid | Reduced retinal thickness compared to the previous examination (baseline) with no signs of significant accumulation of intra- or subretinal fluid |
| **CASE-03** | **Day 0** | **Day 15** | **Day 45** |
| **Symptoms** | Decreased visual acuity in the left eye | Decreased visual acuity in the left eye | Decreased visual acuity in the left eye |
| **Visual acuity** | Right eye = 20/20  Left eye = 20/40 | Right eye = 20/20  Left eye = 20/40 | Right eye = 20/20  Left eye = 20/40 |
| **Tonometry** | Right eye = 12mmHg  Left eye = 12mmHg | Right eye = 12mmHg  Left eye = 12mmHg | Right eye = 12mmHg  Left eye = 12mmHg |
| **Anterior segment Biomicroscopy** | Cells in the anterior chamber 1+/4+  (fine keratic precipitates) | Cells in the anterior chamber 1+/4+  (fine keratic precipitates) | Cells in the anterior chamber 1+/4+  (fine keratic precipitates) |
| **Posterior segment Biomicroscopy** | Vitreous cells 2+/4+  Vitreous haze 1+/4+ | Vitreous cells 2+/4+  Vitreous haze 1+/4+ | Vitreous cells 2+/4+  Vitreous haze 1+/4+ |
| **Fundus photography** | Satellite retinochoroiditis lesion in the upper peripheral media of the left eye by a healed retinochoroiditis lesion (old appearance) | Satellite retinochoroiditis lesion in the upper peripheral media of the left eye by a healed retinochoroiditis lesion (old appearance) | Satellite retinochoroiditis lesion in the upper peripheral media of the left eye by a healed retinochoroiditis lesion (old appearance) |
| **Fluorescein angiography** | Progressive hyperfluorescence with delayed leakage of contrast corresponding to active lesion and masked hypofluorescence corresponding to the old scarred lesion | Progressive hyperfluorescence with delayed leakage of contrast corresponding to active lesion and masked hypofluorescence corresponding to the old scarred lesion | Progressive hyperfluorescence with delayed leakage of contrast corresponding to active lesion and masked hypofluorescence corresponding to the oldscarred lesion |
| **Optical coherence tomography (OCT)** | Bad quality of OCT examination due to lesion location | Bad quality of OCT examination due to lesion location | Bad quality of OCT examination due to lesion location |
| **CASE-04** | **Day 0** | **Day 15** | **Day 45** |
| **Symptoms** | Decreased visual acuity in the right eye | Partial improvement of symptoms | Reported significant improvement in visual acuity |
| **Visual acuity** | Right eye = 20/40  Left eye = 20/20 | Right eye = 20/25  Left eye = 20/20 | Right eye = 20/20  Left eye = 20/20 |
| **Tonometry** | Right eye = 12mmHg  Left eye = 12mmHg | Right eye = 12mmHg  Left eye = 12mmHg | Right eye = 12mmHg  Left eye = 12mmHg |
| **Anterior segment Biomicroscopy** | Cells in the anterior chamber 1+/4+  (fine keratic precipitates) | No cells in the anterior chamber Reduction in keratic precipitates | No cells in the anterior chamber No keratic precipitates |
| **Posterior segment Biomicroscopy** | Vitreous cells 2+/4+  Vitreous haze 2+/4+ | Vitreous cells 1+/4+  Vitreous haze 1+/4+ | No Vitreous cells  No vitreous haze |
| **Fundus photography** | Satellite retinochoroiditis lesion in the upper peripheral media of the right eye beside of a healed retinochoroiditis lesion (old appearance) | Healing satellite retinochoroiditis lesion in the upper peripheral media of the right eye beside of a healed retinochoroiditis lesion (old appearance) | Healed satellite retinochoroiditis lesion in the upper peripheral media of the right eye beside of a healed retinochoroiditis lesion (old appearance) |
| **Fluorescein angiography** | Progressive hyperfluorescence with delayed leakage of contrast corresponding to active lesion and masked hypofluorescence corresponding to the old scarred lesion | Delayed discreet ‘staining’ characteristic of healing of lesion | Blocked hypofluorescence corresponding to lesion without areas of delayed leakage or ‘staining’ of the contrast |
| **Optical coherence tomography (OCT)** | Bad quality of OCT examination due to lesion location | Bad quality of OCT examination due to lesion location | Bad quality of OCT examination due to lesion location |
| **CASE-05** | **Day 0** | **Day 15** | **Day 45** |
| **Symptoms** | Decreased visual acuity in the left eye | Partial improvement of symptoms | Reported significant improvement in visual acuity |
| **Visual acuity** | Right eye = 20/25  Left eye = 20/150 | Right eye = 20/25  Left eye = 20/40 | Right eye = 20/25  Left eye = 20/40 |
| **Tonometry** | Right eye = 15mmHg  Left eye = 14mmHg | Right eye = 15mmHg  Left eye = 15mmHg | Right eye = 15mmHg  Left eye = 15mmHg |
| **Anterior segment Biomicroscopy** | Cells in the anterior chamber 2+/4+  (granulated keratic precipitates) | Cells in the anterior chamber 1+/4+ Reduction in keratic precipitates | No Cells in the anterior chamber  Few precipitates |
| **Posterior segment Biomicroscopy** | Vitreous cells 3+/4+  Vitreous Haze 3+/4+ | Vitreous cells 2+/4+  Vitreous haze 2+/4+ | Vitreous cells 1+/4+  Vitreous haze 1+/4+ |
| **Fundus photography** | Satellite retinochoroiditis lesion temporal to the macula region of the left eye beside a scarred retinochoroiditis lesion de (old appearance) | Satellite retinochoroiditis lesion temporal to the macula region of the left eye beside a scarred retinochoroiditis lesion (old appearance) | Retinochoroiditis lesion temporal to the macula region of the left eye beside a scarred retinochoroiditis lesion de (old appearance) |
| **Fluorescein angiography** | Progressive hyperfluorescence with delayed leakage of contrast corresponding to active lesion and masked hypofluorescence corresponding to the old scarred lesion | Delayed discreet ‘staining’ characteristic of healing of lesion | Blocked hypofluorescence corresponding to lesion without areas of delayed leakage or ‘staining’ of the contrast |
| **Optical coherence tomography (OCT)** | Increased retinal thickness temporal to the macula.  Analysis made difficult by the vitreous haze | Increased retinal thickness temporal to the macula.  Analysis made difficult by the vitreous haze | Reduced retinal thickness temporal o the macula |
